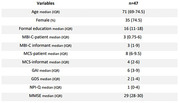# Mild Behavioral Impairment in individuals with subjective cognitive decline and mild cognitive impairment in Southern Brazil

**DOI:** 10.1002/alz.093065

**Published:** 2025-01-03

**Authors:** Victória Tizeli Souza, Simone Sieben da Mota, Gabriela Raquel Rivas Paz, Bruno De Oliveira De Marchi, Guilherme Da Silva Carvalho, Haniel Bispo De Souza, Lucas Bastos Beltrami, Rhaná Carolina Santos, Sarah Vitoria Bristot Carnevalli, Ana Letícia Amorim de Albuquerque, Leonardo Martins de Paula, Manuella Edler Zandoná Giordani, Wyllians Vendramini Borelli, Giovanna Carello‐Collar, Marcia L Fagundes Chaves, Eduardo R. Zimmer, Raphael Machado Castilhos

**Affiliations:** ^1^ Hospital de Clínicas de Porto Alegre, Porto Alegre, Rio Grande do Sul Brazil; ^2^ Universidade Federal Rio Grande do Sul, Porto Alegre Brazil; ^3^ Universidade Federal do Rio Grande do Sul, Porto Alegre, RS Brazil; ^4^ Universidade do Vale do Rio dos Sinos, São Leopoldo, Rio Grande do Sul Brazil; ^5^ Universidade Federal de Santa Catarina, Florianópolis Brazil; ^6^ Clinical Hospital of Porto Alegre, Porto Alegre, Rio Grande do Sul Brazil; ^7^ Universidade Federal do Rio Grande do Sul, Porto Alegre Brazil

## Abstract

**Background:**

Mild Behavioral Impairment (MBI) is a construct developed to capture neuropsychiatric symptoms in individuals in early stages of neurodegenerative diseases. The assessment of MBI in individuals with preclinical cognitive manifestations in Brazil is still quite limited. The aim of this study is to evaluate the MBI‐Checklist (MBI‐C) in individuals with subjective cognitive decline (SCD) and mild cognitive impairment (MCI) in the Brazilian Subjective Cognitive Decline (BRASCODE) cohort in southern Brazil.

**Method:**

BRASCODE cohort is currently following cognitive unimpaired individuals with > 60 years with cognitive complaints and without severe clinical or neuropsychiatric illness. In this analysis we included the individuals evaluated in the 12 months follow‐up. We performed the following scales: MBI‐C (participant and informant), Neuropsychiatric Inventory–Questionnaire (NPI‐Q), Memory Complaint Scale (MCS) (participant and informant), Geriatric Anxiety Inventory (GAI), Geriatric Depression Scale (GDS), and Mini Mental State Examination (MMSE). A correlation analysis was conducted among these variables.

**Result:**

Until December 2023, 47 individuals performed the 12‐month evaluation. Table 1 described the characteristics of the participants. The MBI‐C of the patient and informant had medians (IQR) of 3(0.75‐6) and 3(1‐9), respectively. The MBI‐C‐informant showed a strong positive correlation with the NPI (p‐value<0.0001, rho = 0.827) and also correlated with the SCD‐informant (p‐value = 0.006, rho = 0.404). The MBI‐C‐patient correlated positively with GDS (p‐value<0.0001, rho = 0.604) and with GAI (p‐value = 0.049, rho = 0.314), and negatively with their education (p‐value = 0.009, rho ‐0.387), but it was not with NPI, MCS‐patient and MCS‐informant Particularly, MBI‐C of the participant and informant did not correlate.

**Conclusion:**

Our partial analyses showed that informant perceptions of neuropsychiatric and memory manifestations were correlated in participants with SCD and MCI. In addition, MBI‐C of the participants was correlated with anxiety and depression symptoms. Studies with a larger sample size may provide additional information about MBI in individuals with SCD and MCI.